# Sensitive Organic Vapor Sensors Based on Flexible Porous Conductive Composites with Multilevel Pores and Thin, Rough, Hollow-Wall Structure

**DOI:** 10.3390/polym14224809

**Published:** 2022-11-09

**Authors:** Ting-Ting Kong, Jia-Hai Zhou, Feng Nie, Chao Zhang, Fei-Xiang Shen, Shou-Wei Dai, Hong-Tao Pan, Li-Xiu Gong, Li Zhao

**Affiliations:** 1 College of Material, Chemistry and Chemical Engineering, Key Laboratory of Organosilicon Chemistry and Material Technology of Ministry of Education, Hangzhou Normal University, Hangzhou 311121, China; 2 Zhejiang Chuanhua Chemical Group Co., Ltd., Hangzhou 311215, China

**Keywords:** polydimethylsiloxane, porous conductive polymer composites, vapor grown carbon nanofiber, organic vapor-sensing behavior, sensitivity

## Abstract

Advanced organic vapor sensors that simultaneously have high sensitivity, fast response, and good reproducibility are required. Herein, flexible, robust, and conductive vapor-grown carbon fibers (VGCFs)-filled polydimethylsiloxane (PDMS) porous composites (VGCFs/PDMS sponge (CPS)) with multilevel pores and thin, rough, and hollows wall were prepared based on the sacrificial template method and a simple dip-spin-coating process. The optimized material showed outstanding mechanical elasticity and durability, good electrical conductivity and hydrophobicity, as well as excellent acid and alkali tolerance. Additionally, CPS exhibited good reproducible sensing behavior, with a high sensitivity of ~1.5 × 10^5^ s^−1^ for both static and flowing organic vapor, which was not affected in cases such as 20% squeezing deformation or environment humidity distraction (20~60% RH). Interestingly, both the reproducibility and sensitivity of CPS were better than those of film-shaped VGCFs/PDMS (CP), which has a thickness of two hundred microns. Therefore, the contradiction between the reproducibility and high sensitivity was well-solved here. The above excellent performance could be ascribed to the unique porous structures and the rough, thin, hollow wall of CPS, providing various gas channels and large contact areas for organic vapor penetration and diffusion. This work paves a new way for developing advanced vapor sensors by optimizing and tailoring the pore structure.

## 1. Introduction

Gas sensors that convey information and early warnings about flammable, harmful, and dangerous gases leaking into environment have attracted tremendous interest due to their broad applications in the fields of security, health, environmental monitoring, industry, medical applications, and so forth [1,2,3,4,5,6]. Among them, conductive polymer composites (CPCs), which are composed of a polymer matrix and conductive fillers, have been considered suitable candidates as gas sensors due to their low cost, ease of fabrication, and good environmental stability [2,7,8]. The most widely accepted fundamental of CPCs is this: when immersed in vapors, the adsorption of organic vapors and, hence, the swelling of the polymer matrix cause a break in the filler network and increase the resistance; finally, the damaged conductive paths recover to their original states after vapor desorption [9,10]. Ideal gas sensors always require high sensitivity, a fast response time, excellent repeatability, and wide applicability.

It has been found that the performance of CPC gas-sensitive materials is closely related to the structure of polymers [11], affinity between the polymer matrix and target gas [12,13], types and morphology of conductive fillers [10,14], structure of conductive particle network [9,15], and so on. In the past several decades, much work has been devoted to improving sensing performance by designing conductive network architectures; e.g., the responsivity of CPC vapor sensors can be enhanced by the surface modification of the fillers [16,17,18,19]. The reconstruction and stability of the conductive network can be enhanced by processing optimization [13,20] and cross-linking treatment of the polymer [21], thus improving the reproducibility of the sensing behavior. A series of works has been conducted to study the effect of the morphology and type of carbon nanoparticles. Generally, hybrids of particles with different dimensions can optimize the sensing performance. For example, the gas-sensing response behavior of composites containing one-dimensional (1D) carbon fillers (carbon nanotubes and carbon nanofibers) is more stable and recoverable than that of the composites filled only with traditional and spherical carbon black (CB), as bridge-like conductive networks are better and more stable than point–point contact networks among spherical particles [14,22]. However, it is still a challenge to construct CPC sensors that simultaneously have high sensitivity, a fast response, good reproducibility, and wide applicability. For example, although the stability and reproducibility can be optimized by thee cross-linking treatment of the polymer matrix, the swelling degree is sacrificed, resulting in a decrease in responsiveness. Although tailoring the conductive network using second fiber-shaped fillers improved the sensing stability of CPCs, the sensitivity was inevitably weakened [23].

The shape of a gas-sensitive material is also a key factor affecting its performance [20,24,25,26]. A rapid response requires a senor material with a large specific surface area, so that the gas can quickly permeate into and then swell the material. CPC gas-sensing materials are usually prepared as thin films, where the thinner the film, the faster the responsive speed [7,20,27]. However, the film-forming process becomes very difficult due to the viscosity and modulus of the composite fluid significantly increasing with the addition of high contents of fillers. Generally, the thickness of common conductive CPC films is limited to tens to hundreds of micrometers. Due to the weak strength and limited thickness of CPC films, the development of new generations of high-performance gas sensors has reached a bottleneck. Comparatively, robust three dimensional (3D) porous conductive materials, with their large surface area and abundant pore structure, have great potential in gas sensing [24,28,29,30,31].

In this study, a simple strategy was developed to fabricate high-performance, flexible, 3D, sponge gas sensors via the construction of an abundant, multilevel, through-pores structure and elastic, conductive, thin, and hollow walls to expand the specific surface area and achieve high permeability. We chose a cross-linking chain structure, polydimethylsiloxane (PDMS) silicone rubber, which has high elasticity, excellent high- and low-temperature resistance, and good hydrophobicity as the matrix [32,33]. Advanced one-dimensional vapor-grown carbon fibers (VGCFs) were selected to construct an easily variable and well-recoverable bridge-like conductive network. The VGCFs-filled PDMS porous composites (VGCFs/PDMS sponges, CPSs) were synthesized simply via coating a VGCFs/PDMS (CP) thin layer on sacrificial nickel foam skeletons, which was combined with the wall structure (thickness and irregular pore on the wall) that was further tailored by adjusting the concentration of the coating suspension and applying centrifugal process conditions. The relationships between material structure–mechanical properties–gas-sensitive behaviors were carefully investigated. The optimized CPSs had an extraordinary multilevel pore structure: the first interconnective pores, with a large size of ~600 µm, were similar to Ni foam’s pores; the second-level pores (hollow skeleton) formed after the Ni skeleton was etched away; and the third-level pores (large number of irregular pores) were located on the thin, conductive VGCFs/PDMS wall. Such 3D porous composites presented not only good mechanical elasticity and structural stability, but also superior gas-sensing properties such as rapid response time, high sensitivity, and good reproducibility, which we reliably operated under flowing vapors and high-humidity disturbance conditions. Finally, the related sensing mechanisms were analyzed and proposed.

## 2. Experimental Section

### 2.1. Materials

VGCFs, with an average diameter of 150 nm, length of 10 μm, and electrical resistivity of 1 × 10^−4^ Ω cm, were purchased from Showa Denko Co. Ltd., Tokyo, Japan. Commercial Ni foam, with pore size of 400~800 μm, was purchased from Kunshan Jiayisheng Electronics Co., Ltd., Kunshan, China. PDMS (Sylgard 184 silicone rubber base) and curing agent were bought from Dow Corning, Midland, the United State. Inhibitor was bought from Changzhou Dongshun Chemical Co., Ltd., Changzhou, China. Analytical-grade solvents, such as xylene, ethyl alcohol, carbon tetrachloride, ethyl acetate, n-hexane, cyclohexane, and acetone, were supplied by Sinopharm Chemical Reagent Co., Ltd., Shanghai, China. All the chemicals were used as received without any further purification.

### 2.2. Preparation of Film-Shaped Composites

The film-shaped composites were prepared by the following steps: First, the dried VGCFs were added into xylene and premixed for 20 min by sonication. Subsequently, PDMS, together with curing agent and inhibitor, was added to the above VGCFs/xylene suspension and then stirred at 2000 rpm for 30 min to obtain the VGCFs/PDMS/xylene suspension (xylene/PDMS ratio of 5:1). Two common processes (solution casting and melt-molding) were used to prepare the film-shaped samples. The solution casting process was as follows: the suspension was poured into a Petri dish and placed in a fume hood for 12 h to evaporate a large amount of solvent, which was then transferred to a drying oven (60 °C, 5 h) to fully evaporate the residual xylene, and was then finally cured at 120 °C for 2 h. The thickness of the film was regulated according to the amount of suspension agent. The hot-molding process was operated after removing the xylene solvent as above. Then, the obtained viscous sample was pressed into a film at 120 °C in a flat vulcanizer, and the PDMS was cured while the film was pressed.

### 2.3. Preparation of Porous Composites

The CPS composites were prepared according to the following procedure: Ni foam (2 × 2 × 1 cm^3^) was ultrasonically washed in ethanol and acetone several times to remove the oxides and dust from the surface and dried in a blast oven at 80 °C for 20 min. After that, the cleaned Ni foam was dipped into the pre-prepared CP/xylene suspension (preparation steps were the same as before, with a xylene/PDMS ratio of 5:1), and the pores were filled with CP suspension. Then, the obtained foam was taken out to exhaust any bubbles under a vacuum. After that, the Ni foams were spin-coated (600 rpm, 1 min) to remove excess CP mixture, and the obtained CP/xylene-coated Ni foam composites were then dried in an oven to remove the residual xylene and cured PDMS (120 °C, 2 h). Finally, the obtained composite was soaked in a 4 mol/L hydrochloric acid solution for several days to etch away the nickel foam, and the composite was washed with water and then dried for further usage.

### 2.4. Characterization

#### 2.4.1. General Techniques

The morphology and structure of the prepared samples were characterized by scanning electron microscopy (SEM) (Sigma-500, ZEISS, Oberkochen, Germany). Before observation, the samples were sputtered with gold for 30 s. The compressive properties of the CPSs were measured using a universal testing machine (Lloyd Instrument LS100, Ametek, America). The dynamic viscoelastic properties of different content of VGCFs/PDMS prepolymers were characterized by a rheometer (DHR-2, TA instruments, Newcastle, America). The measurements were carried out at 30 °C under small amplitude oscillatory shear mode using parallel plate geometry (25 mm diameter). Frequency sweeps ranging from 100 to 0.02 rad/s were used at a strain of 0.05%. The water contact angle (WCA) of the CPS sample was measured with a DSA30 CA analyzer (Kruss, Hamburg, Germany) with a 3 μL water droplet. Assisted by silver paste, electrodes (two pieces of copper meshes) were carefully attached to the opposite surface the sample, then a two-probe resistance measurement was carried out to determine the resistance of samples with a digital multimeter (Escort-3146 A, Schmidt Scientific Taiwan Ltd., Taiwan, China). A high-resistance meter (ZC36, Shanghai Jingke Industrial Co. Ltd., Shanghai, China) was used to characterize the initial high resistance of the VGCFs/PDMS composite samples containing a low VGCF concentration. The resistivity of the VGCFs/PDMS composites was calculated using ρ = RS/L, where ρ (Ω∙cm) is the resistivity; R (Ω) is the electrical resistance and L (cm) and S (cm^2^) are the length and cross-section area of the composites, respectively. 

#### 2.4.2. Characterization of Vapor-Sensing Behaviors

The vapor-sensing behaviors of the static saturated organic vapor of the samples were investigated using a device, as illustrated in Figure S1a [34]. The material was alternately placed in a container containing n-hexane solvent for 100 s and in air for 200 s, and the real time-resistance values were recorded with a source meter (2601B, Keithley, Solon, America).

The low-concentration-detection capability of sensing behavior was tested using another homemade device (Figure S1b) [29]. The sample was connected to a source meter and placed in a container. By injecting a certain weight of n-hexane solvent into the container, the n-hexane solvent quickly volatilized and filled the entire test container. Real-time resistance values were recorded during the cyclic changes of the container atmosphere: organic vapor with a specified low concentration (100 s) to dry air (100 s). To obtain a stable baseline, the resistances of all the samples were recorded for 50 s under dry air before starting the measurement. Samples were tested at 25 °C and ~20% RH.

The experimental device for detecting the sensing performance for flowing organic vapor is shown in Figure S1c [35]. A bubbler evaporation system was utilized to deliver a controlled concentration of organic gas to the test chamber using dry air as the carrier and diluting gas. The temperature of the bubbler evaporation system was set to 25 °C. The total flow rate was kept constant at 500 cm^3^ min^−1^ by mass flow controllers (MFCs) during the measurements. The concentration of the analyte, which was carefully controlled by two MFCs, was calculated by Equation (1):

(1) $$Con_{Vapor}\left(\%\right)=\left(\frac{P_{i}}{P}\times \frac{f_{1}}{f_{1}+f_{2}}\right)\times 100$$

(2) $$\mathrm{Lg}P_{i}=A-\frac{B}{C+T}$$

In Equation (1), *f*_1_ and *f*_2_ are the mass flow rate of MFC 1 and MFC 3, respectively; P is the standard atmospheric pressure; and Pi is the saturated partial pressure of solvent empirically obtained by the Antoine equation (Equation (2)) (mmHg), where *A*, *B*, and *C* are Antoine constants, and *T* is the temperature (°C).

## 3. Results and Discussion

### 3.1. Fabrication and Structural Characterization of Porous CPS Composites

**Figure 1:**
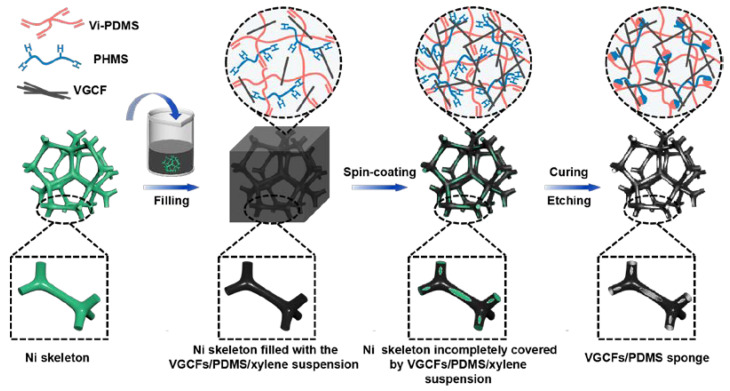
Fabrication schematic of VGCFs/PDMS sponge (CPS) via applying the dip-spin-coating method.

A schematic of the fabrication process of the porous CPS composite is illustrated in Figure 1. The main steps for the preparation of the organic vapor sensors are preparation of a conductive CP suspension with good filler dispersion and fabrication with a designed porous structure included a dip-spin-coating method using Ni sponge as the sacrificial template [36,37]. Herein, CP suspensions with a VGCF dispersion were firstly prepared by the solution blending method, and then the CP prepolymer and CP films were prepared to study the influence of VGCF contents on the dynamic viscoelastic, electrical, and gas-sensing properties of the composites to determine the appropriate VGCF content. The corresponding results are shown in Figure S2. The typical viscosity percolation (3.5~8 vol%) and electrical percolation threshold (1.8~7 vol%) well-indicated that the conductive chains had formed and then assembled into a conductive network with further increases in the filler content. When the content of VGCFs was equal to 10 vol%, liquid-to-solid transition (*G*’ > *G*’’) (Figure S2a) appeared, which was assigned to the modeled filler networking. According to the above results, the CP composites with VGCF contents above the percolation threshold (8, 10, and 12 vol% VGCFs) were chosen to test the gas-sensing and typical response behaviors of these composites for n-hexane vapor, as shown in Figure S2d. Among them, the CP composites containing 10 vol% VGCF showed the best vapor-sensing behavior, fastest response, and best recoverability. Consequently, the appropriate VGCF content was 10 vol% here. Next, the Ni foam was filled with the CP/xylene suspension by applying dipping process and further tailoring the coating content, coating layer thickness, and so on, with a spin process. The CP/Ni sponge composite with CP suspension coated onto the partial area of the Ni-skeleton surface was prepared using a xylene/PDMS ratio of 5:1 under the spin-coating condition of 600 rpm for 1 min. Then, the CP-suspension-coated Ni sponge was dried and cured in a hot oven. Finally, the CPS was prepared by Ni-skeleton etching. 

**Figure 2:**
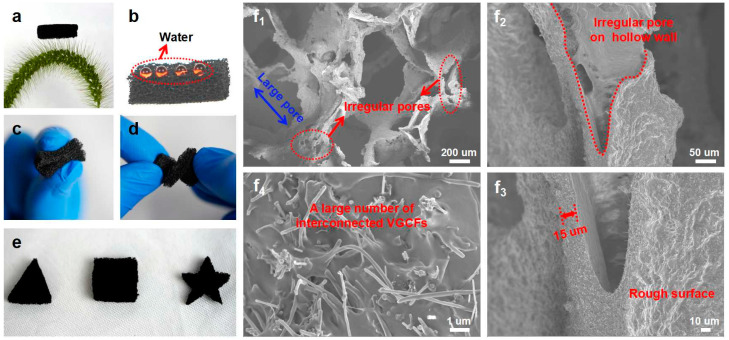
(**a**) Photograph of CPS standing on the bristle grass without bowing any hairy branches. (**b**) Photograph of water droplets on the surface of the VGCFs/PDMS sponge. (**c**,**d**) Flexibility of the CPS. (**e**) Photograph of CPS samples with different patterns. (**f_1_**) SEM images of CPS and its hollow skeleton (**f_2_**,**f_3_**) and the cross-section of CP wall (**f_4_**).

As shown in Figure 2a–d, the resultant sample was ultra-lightweight (standing on bristle grass without bowing any hairy branches), robust, and flexible. It could be bent and twisted without fracture. Different patterns, such as triangles, squares, and five-pointed stars, could be obtained by cutting (Figure 2e). Moreover, the sample had good hydrophobicity (Figure 2b). The morphology and microstructures of the above CPS were carefully observed by SEM, and are shown in Figure 2f. As expected, the CPS composite had abundant pores: big pores of the similar size to those of the Ni-sponge template, with a hollow structure after etching of the Ni skeletons, and many irregular pores on these hollow skeleton walls (as denoted by the blue and red marks in Figure 2f_1_,f_2_). It should be noted that the surface of the skeleton was very rough with up-and-down wrinkles and some interconnected VGCF segments protruding from the wall surface, and the thickness of the thin walls was only 10~20 microns (Figure 2f_3_). The high-magnification SEM images of the fractured cross-section surface showed that the VGCFs randomly dispersed and connected with each other to form bridge-like conductive networks (Figure 2f_4_). The samples had low resistance of ~2 kΩ (Figure S3b). It is worth noting that the porous composites with such a unique structure proved their feasibility as an advanced gas sensor: the highly porous network structure provides many channels for vapor, and the high surface area and thin wall are beneficial for vapor immersion. 

### 3.2. Mechanical Properties and Structural Stability

**Figure 3:**
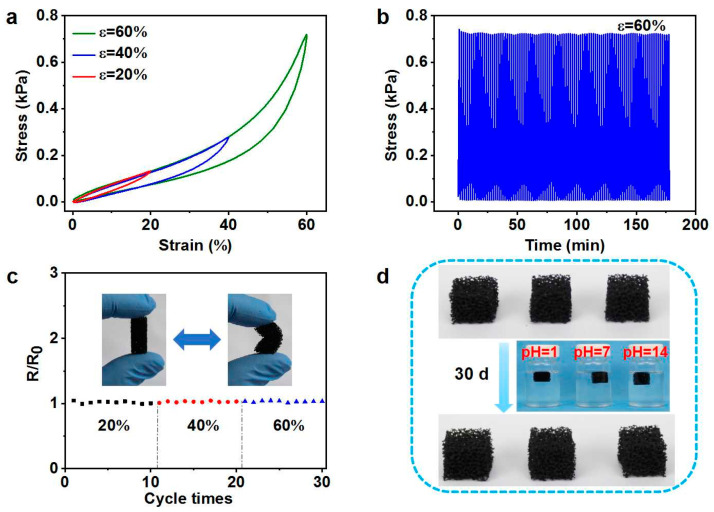
(**a**) Stress–strain curves of CPS at maximum strains of 20%, 40%, and 60%. (**b**) Cyclic compressive test of CPS at 60% strain for 100 cycles. (**c**) Electrical resistance change of CPS after cyclic bending to different degrees. (**d**) Structural stability of CPS after immersion into pH = 1, 7, and 14 solutions for 30 days.

For practical applications, the material must have a certain strength. In view of this, compressive measurements of CPS were carried out, and the obtained compressive stress-strain behaviors under different strains are shown in Figure 3a. The maximum compressive stress of CPS was ~0.7 kPa at 60% strain, which is comparable to that of soft polyurethane sponge [38]. The curves contained the three typical stages of cellular sponges: a linear elastic region for the bending of the structures, a plateau region for buckling phenomena, and a steep-slope region attributed to the densification of sponges. The stress returned to its initial value after unloading for each strain, indicating the good resilience of the composite. Moreover, the maximum stress values at ε = 60% of the samples were almost constant, even after 100 cycles (Figure 3b), demonstrating the stable mechanical flexibility and fatigue resistance. This outstanding structural stability was also proven by its well-maintained initial resistance (R/R_0_ ≈ 1) after bending the sample several times to different states (20%, 40%, and 60%), as shown in Figure 3c. The outstanding flexibility and robustness of the composite could be mainly attributed to its interconnected porous structure, the excellent mechanical properties of the cross-linking silicone rubber (SR), and the reinforcing effect by the VGCFs of the matrix, which guaranteed its reliability for repeated and long-term usage. In addition, it can be clearly seen that the composites showed excellent acid and alkali resistance when the samples were immersed into acid (pH = 1), aqueous (pH = 7), and alkali (pH = 14) solutions for 30 days (Figure 3d).

### 3.3. Static Organic Vapor-Sensing Behaviors

**Figure 4:**
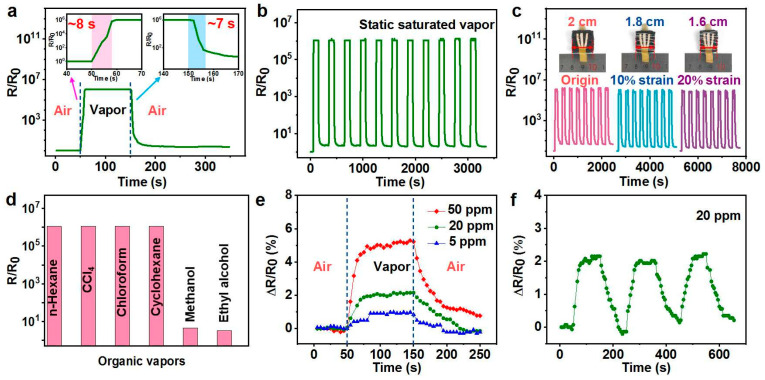
(**a**) Vapor-sensing behavior of CPS to static saturated n-hexane vapor. The inset shows the response and recovery times of CPS. (**b**) Cyclic vapor-sensing behavior of CPS toward saturated n-hexane vapor. (**c**) Responsivity of CPS maintained at different compressions. (**d**) Maximum responsivity of CPS to different saturated volatile organic vapors. (**e**) Gas-sensing results of CPS for n-hexane vapor with low concentrations (5, 20, and 50 ppm). (**f**) Gas-sensing results of CPS to 20 ppm of n-hexane vapor in successive vacuum–vapor–vacuum cycles.

A typical vapor-sensing behavior, when exposed to a saturated vapor (n-hexane) of the resulting CPS, is shown in Figure 4a. The R/R_0_ obviously increased to the maximum values when the sample was exposed to static saturated n-hexane vapor. Then, the changed resistance rapidly recovered to almost the initial value once in dry air. The responsivity (R/R_0_), response/recovery time (t_response/_t_recovery_), and the sensitivity, which comprehensively reflect the parameters of response time and responsivity, are the crucial indices for resistance-type vapor sensors [39]. The porous CP composite had a high responsivity of 10^6^, and response and recovery times of ~8 s and ~7 s, respectively. In addition, the CPS showed outstanding cyclic performances (Figure 4b). After the second cycle, all the resistances increased or decreases to almost the same level in alternating n-hexane and dry air in the following ten cycles. Here, the sensitivity (S = (R_max_/R_0_)/t_response_) of the sensor is defined as the maximum responsivity (R_max/_R_0_) versus the corresponding time (t_response_). After calculation, the sensitivity of CPS to static saturated n-hexane vapor was as high as 1.5 × 10^5^ s^−1^. Clearly, the CPS presented comparable sensitivity to film-shaped CP with a thickness of 20 μm, and better sensing performance than that of CP films with thicknesses of 50 and 200 μm due to faster response and better recovery capability (Figure S4b–d). However, such thin films (20 and 50 μm) are difficult to prepare even using solution casting or hot-pressure molding and are unsuitable for further use due to their low strength (Figure S4a_1_,a_2_). Increasing its thickness to strengthen the film would weaken the sensing performance (Figure S4c,d). Therefore, the robust and good permeability of 3D porous structures are quite suitable for constructing ideal-gas-sensitive materials. Fortunately, the sensing behaviors of the CPS were also better than those for previously reported CPCs (Table S2) [14,40]. It is worth noting that the sensing response behavior was hardly affected when the material was subjected to a 20% compressive deformation due to its abundant interconnected gas channels (Figure 4c).

In our work, a series of solvents was selected to evaluate vapor-sensing selectivity. When exposed to n-hexane, carbon tetrachloride, chloroform, and cyclohexane for 100 s, the responsivity of samples all reached ~10^6^ in a short time (Figure 4d and Table S1). The R/R_0_ of the composites increased little in methanol and ethanol (Figure 4d). As mentioned before, the responsivity is closely related to the swelling degree of the polymer matrix, which is determined by the interaction between the solvent and polymer [13]. The physical properties of these solvents are shown in Table S1. The interaction between the solvent and polymer could be evaluated using the Flory–Huggins interaction parameter χ_12_, which we calculated according to Equation (3), and the corresponding values for solvents are recorded in Table S1.

(3) $$\chi_{12}=\frac{V_{0}{\left(\delta_{1}-\delta_{2}\right)}^{2}}{RT}$$

In Equation (3), *V*_0_ refers to the molar volume of the solvent (cm^3^/mol); *T* represents the temperature (K); *R* is the ideal vapor constant (8.314 J/mol); and δ1 and δ2 are the solvent and polymer solubility parameters (J^1/2^/cm^3/2^), respectively. The solubility parameter value of PDMS is ~15.3 (J^1/2^/cm^3/2^). In theory, a low χ_12_ means a high solubility of the solvent to the polymer, which corresponds to high sensitivity. Thus, when the χ_12_ is lower, more severe damage to the VGCF network leads to a higher electrical responsivity. For example, the maximum responsivity of porous CPS composite for n-hexane (χ_12_ = 0.008) was ~1.2 × 10^6^.

The low-concentration-detection capability is also a very important indicator. The changes in the relative resistance (ΔR/R_0_, where ΔR = R − R_0_, R_0_ stands for the original resistance, and R is the resistance during the test) to three low concentrations (5, 20, and 50 ppm) of n-hexane vapor are displayed in Figure 4e. Change in resistance could be observed even under a 5 ppm vapor. Furthermore, we conducted a cyclic response–recovery experiment for CPS to 20 ppm n-hexane vapor. The sensing curves of each cycle were similar, indicating good recyclability in detecting low-concentration vapors (Figure 4f).

### 3.4. Flowing Organic Vapor-Sensing Behaviors

**Figure 5:**
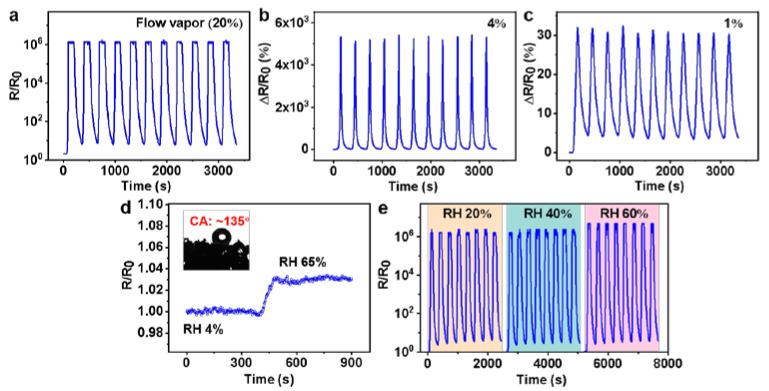
(**a**) Cyclic vapor-sensing behavior of CPS to flowing, saturated n-hexane vapor. (**b**,**c**) Cyclic vapor-sensing behavior of CPS upon exposure to flowing n-hexane vapor with low concentrations of 4% and 1%, respectively. (**d**) The real-time electrical resistance change of CPS under different RH environments at 25 °C. The inset shows test result of the water contact angle. (**e**) The real-time responsivity of CPS to flowing, saturated n-hexane vapor under different RH environments at 25 °C.

The sensing performance of flowing n-hexane vapor was also studied herein. The concentration of saturated n-hexane (20%) at 25 °C could be calculated by Equations (1) and (2). When valve 2 in Figure S3c was closed, and only valve 1 is opened, the test vapor was the simulated flowing saturated vapor. As shown in Figure 5a, when exposed to flowing saturated n-hexane vapor, the responsivity reached ~1.2 × 10^6^ (similar to the maximum responsivity for the static saturated vapor). Figure 5b,c show the ΔR/R_0_ variation in the CPS composite toward low concentrations (1% and 4%) of flowing n-hexane vapor. The ΔR/R_0_ variation gradually increased with the increase in vapor concentration. The composite also had an obvious response (~5000%) to a concentration of flowing n-hexane vapor as low as 1%. Moreover, the cyclic vapor-sensing behavior remained unchanged even in the low-concentration case. Compared with the work of others, as shown in detail in Table S2, the CPS composite also showed outstanding sensitive performance to flowing organic vapor [26,41].

Humidity is an important environmental factor that cannot be ignored [42]. The inserted photograph in Figure 5d shows the WCA of the sample (~135°), which indicates the hydrophobic ability of the sample. Figure 5d shows the corresponding resistance change in CPS under different relative humidity (RH) environments. The samples exhibited a slight resistance change when the air humidity changed from 4% to 65% RH. Simultaneously, the vapor-sensing performance of flowing, saturated n-hexane vapor at different air humidities (~20% and 60% RH) were tested. As shown in Figure 5e, no obvious change was observed when the air humidity changed from ~20% to 60% RH. The above results indicated the good prospect of CPS as a humidity-resistive gas sensor.

A simple warning system with a light was made to simulate the detection of n-hexane vapor leakage. The gas-sensing device, a light-emitting diode (LED) lamp, and direct-current main constituted the simple series circuit. A LED lamp was used to provide alarm signals. The real-time changes in the light intensity of the LED lamp in subsequent (flowing air → flowing n-hexane vapor → flowing air) runs are displayed in Figure S5a. The LED lamp exhibited high brightness at a lower voltage (8 V) in the initial state, indicating that the sample had good electrical conductivity. Then, we turned on the valve to allow the vapor into the system. It was observed that the brightness of the bulb gradually dimmed and completely extinguished after 30 s (Figure S5b). From this simple alarm device, it can be demonstrated that CPS could effectively monitor organic vapor leakage.

### 3.5. Sensing Mechanism

**Figure 6:**
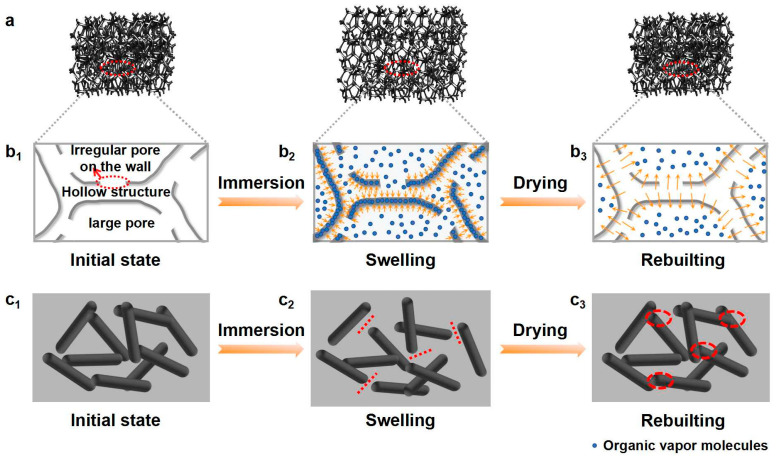
(**a**) Schematic diagram of vapor-penetration- and diffusion-induced swelling behavior of silicone rubber. (**b_1_**–**b_3_**) The process of organic vapor molecules’ penetration and diffusion via multilevel pore structure of CPS, and (**c_1_**–**c_3_**) schematic diagram of conductive network evolution with swelling of SR and then recovery. The red lines and circles depict changes in conductive paths.

The mechanism of the above excellent organic-vapor-sensing behavior is schematically illustrated in Figure 6. CPS has unique structures with multilevel pores (three types of pores: large-size pores, hollow skeleton structure, and many irregular pores on the hollow skeleton wall) and ultra-thin, rough, and hollow wall (Figure 6b_1_). Various interconnected gas channels and large contact areas constructed by the unique pore structure are very beneficial for the fast penetration and diffusion of organic vapors into the composite interior. For example, the vapors can be quickly adsorbed by the big pores and thus contact the outer surface of the skeleton wall or directly entering the interior of the hollow skeletons from the irregular pores. The thin CP wall rapidly swells to a large swelling degree owing to the number of vapor molecules infiltrating the matrix from both the inside and outside surfaces of the thin wall (Figure 6b_2_). Therefore, the destruction of the filler–filler contacts and increase in the interfiber distance by the swelling of the SR matrix severely damaged the conductive network (Figure 6c_2_) and resulted in a rapid and great change in resistance. During drying, organic vapors can also quickly diffuse out through these gas channels; the porous sponge can quickly return to its original state (Figure 6b_3_). Meanwhile, the cross-linking effect of the matrix, combined with the good recoverability of the bridge-like VGCF network, the resistance of the composite almost returned to the initial value (Figure 6c_3_).

## 4. Conclusions

In summary, a flexible and high-performance vapor sensor based on conductive CP sponge was prepared by a dip-spin-coating process using Ni sponge as the sacrificial template. The optimized porous composite has unique structures with an abundant multilevel pore structure (large- pores were similar to those of the Ni sponge template, hollow skeleton structure, and many irregular pores on the hollow-skeleton wall) and a rough and thin skeleton wall. The conductive CPS composites had good elasticity and durability, excellent acid and alkali resistance, and good hydrophobicity. Compared with film-shape composites, the robust porous CP composites exhibited excellent gas-sensing performance, including high sensitivity (~1.5 × 10^5^ s^−1^), rapid response and recovery times (~8/7 s), and good reproducibility. Interestingly, the good sensing behavior was well-maintained even with loading at a 20% strain deformation. So, the previous unsolved problem that reproducibility and sensitivity cannot be achieved at the same time was well-solved here. Moreover, the porous composite exhibited a high sensitivity of 10^5^ s^−1^ to flowing organic vapor. Its cyclic sensing performance was almost not affected by ambient humidity (20~60% RH). The above excellent performance could be ascribed to the unique abundant porous structures with the rough, thin, and hollow wall of CPS providing various connected gas channels and large contact areas for organic gas penetration and diffusion. This work provides a new understanding of the rational design and development of high-performance organic vapor sensors.

## Data Availability

Not applicable.

## Supplementary Materials

The following supporting information can be downloaded at: https://www.mdpi.com/article/10.3390/polym14224809/s1, Figure S1: Device for (a) testing saturated-vapor sensing behavior, (b, c) measuring gas-sensitive behavior to low-concentration solvent vapors and flow solvent vapors, respectively; Figure S2: (a) *G*’ and *G*″ as a function of frequency(*ω*) at 30 °C for unvulcanized VGCFs/PDMS composites. Solid patterns represent *G*’, and the hollow patterns represent *G*″. The measured *G*’ and *G*″ for composites with the VGCFs conten ≥6 vol% are vertically shifted by a factor of 10^n^. (b) |*η**| versus ω at 30 °C for unvulcanized VGCFs/PDMS composites. (c) Electrical resistivity (*ρ*) at room temperature and viscosity at low frequency region (*ω* = 0.02 rad/s) as a function of VGCFs content for CP composites, respectively. (d) Responsivity of CP films (~50 μm) with different VGCFs contents to static saturated n-hexane vapor; Figure S3: (a) SEM images of pristine Ni foam. (b) The weight ratio of CP coating to Ni foam and the resistance of CPS composite; Figure S4: Photographs and corresponding thicknesses of membranes prepared by melt-moulding (a_1_) and solution casting (a_2_), respectively. (b) Vapor sensing behavior of VGCFs/PDMS membranes with different thicknesses to static saturated n-hexane vapor. (c) The relative resistance (R_350_/R_0_) of VGCFs/PDMS membranes with different thicknesses and CPS sample at the time of 350 s. (d) Response/recovery time of VGCFs/PDMS membranes with different thicknesses to static saturated n-hexane vapor; Figure S5: Photographs of simulated detection of n-hexane vapor using CPS composites and the corresponding changes in the brightness of the bulb during one cycle change of flowing vapor and flowing air (a) and in vapor flowing progress (b); Table S1: Solubility parameters, molar volume, Flory-Huggins interaction parameters of target solvents and the corresponding response time of sensing behavior; Table S2: Comparison of maximum responsivity and sensitivity among different vapor sensing materials.
