# Supplementary material for: Sensitive Organic Vapor Sensors Based on Flexible Porous Conductive Composites with Multilevel Pores and Thin, Rough, Hollow-Wall Structure
Source: Polymers (Basel). 2022 Nov 9;14(22):4809. doi: 10.3390/polym14224809 (PMC9697012; doi:10.3390/polym14224809)
Supplement: Supplementary file 1 [file polymers-14-04809-s001.zip › polymers-1990746-supplementary.pdf]

Supplementary Materials for

Sensitive Organic Vapor Sensors Based on Flexible Porous Conductive Composites with Multilevel Pores and Thin, Rough, Hollow-Wall Structure

This file includes:

Figures S1 to S5

Tables S1 to S2

Figure S1. Device for (a) testing saturated-vapor sensing behavior, (b, c) measuring gas-sensitive behavior to low-concentration solvent vapors and flow solvent vapors, respectively.

Figure S2. (a) G' and G'' as a function of frequency(ω) at 30 °C for unvulcanized VGCFs/PDMS composites. Solid patterns represent G', and the hollow patterns represent G''. The measured G' and G'' for composites with the VGCFs conten ≥ 6 vol% are vertically shifted by a factor of 10^n. (b) $|\eta^*|$ versus ω at 30°C for unvulcanized VGCFs/PDMS composites. (c) Electrical resistivity (ρ) at room temperature and viscosity at low frequency region (ω = 0.02 rad/s) as a function of VGCFs content for CP composites, respectively. (d) Responsivity of CP films (~50 μm) with different VGCFs contents to static saturated n-hexane vapor.

Figure S3. (a) SEM images of pristine Ni foam. (b) The weight ratio of CP coating to Ni foam and the resistance of CPS composite.

Figure S4. Photographs and corresponding thicknesses of membranes prepared by melt-moulding (a_1) and solution casting (a_2), respectively. (b) Vapor sensing behavior of VGCFs/PDMS membranes with different thicknesses to static saturated n-hexane vapor. (c) The relative resistance (R_{350}/R_0) of VGCFs/PDMS membranes with different thicknesses and CPS sample at the time of 350s. (d)

Response/recovery time of VGCFs/PDMS membranes with different thicknesses to static saturated n-hexane vapor.

Figure S5. Photographs of simulated detection of n-hexane vapor using CPS composites and the corresponding changes in the brightness of the bulb during one cycle change of flowing vapor and flowing air (a) and in vapor flowing progress (b).

Table S1. Solubility parameters, molar volume, Flory-Huggins interaction parameters of target solvents and the corresponding response time of sensing behavior.

Table S2. Comparison of maximum responsivity and sensitivity among different vapor sensing materials.

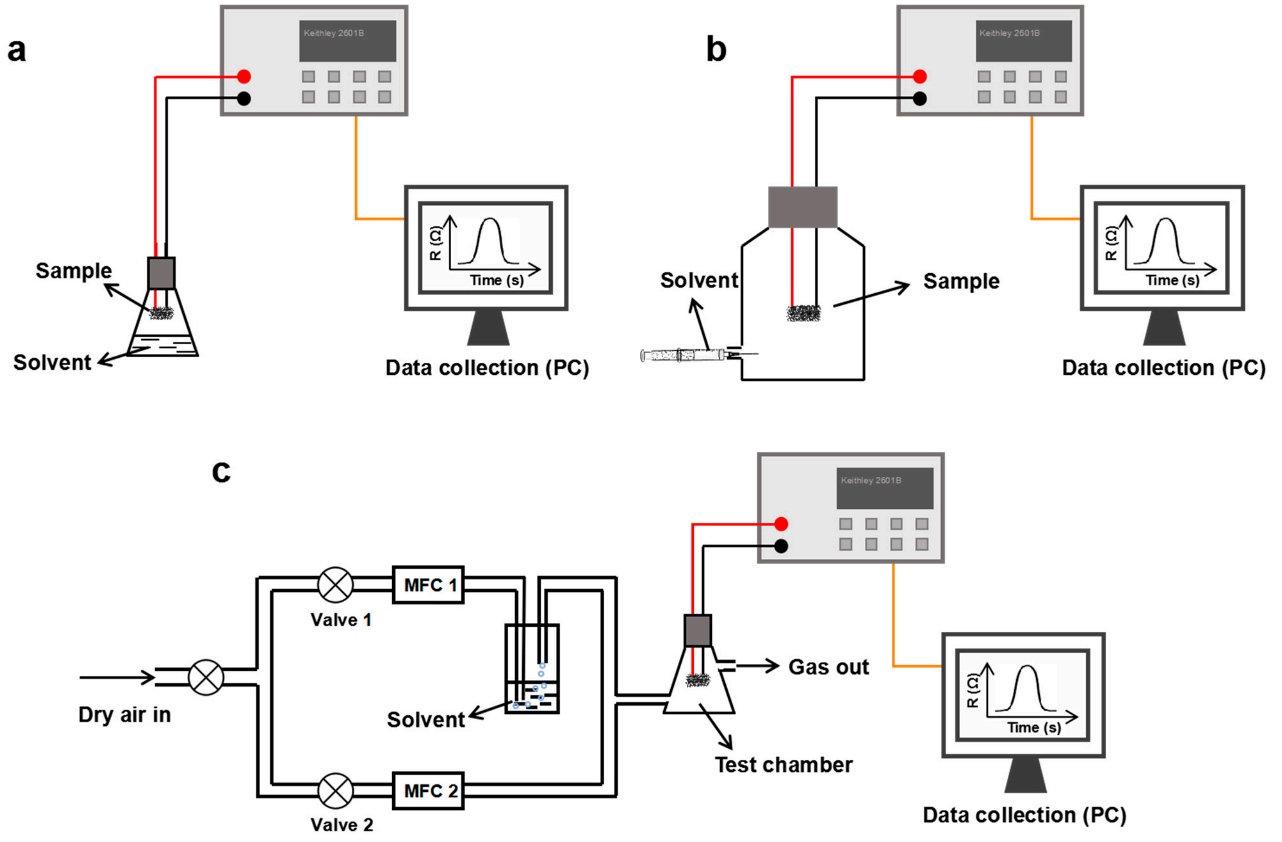

Figure S1. Devices for (a) testing static saturated-vapor sensing behavior, (b) measuring gas-sensitive behavior to low-concentration solvent vapors and (c) testing the sensing behavior to flow solvent vapors, respectively.

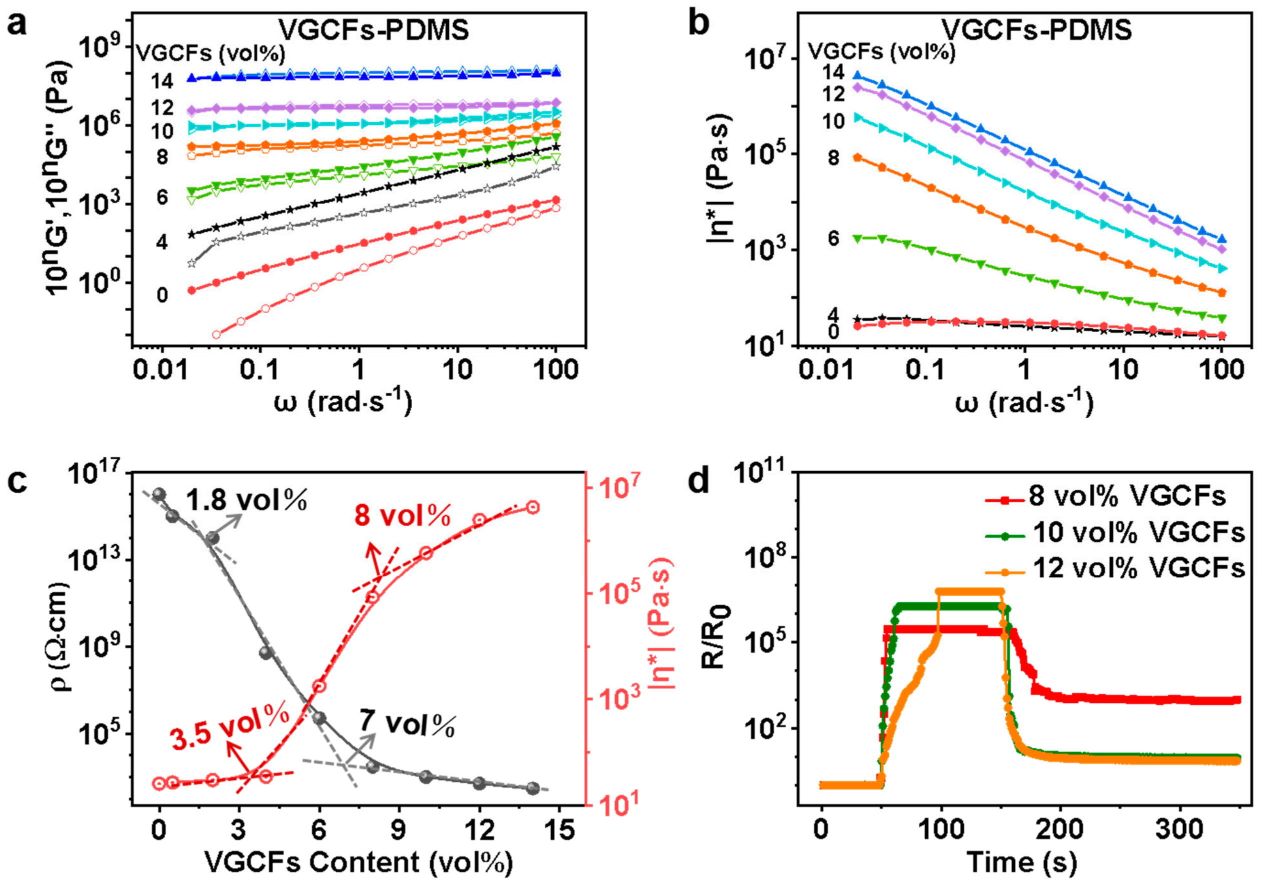

Figure S2. (a) *G*' and *G*″ as a function of frequency(*ω*) at 30°C for unvulcanized VGCFs/PDMS composites. Solid patterns represent *G*', and the hollow patterns represent *G*″. The measured *G*' and *G*″ for composites with the VGCFs content≥6 vol% are vertically shifted by a factor of 10^n. (b) $|\eta^*|$ versus ω at 30°C for unvulcanized VGCFs/PDMS composites. (c) Electrical resistivity (*ρ*) at room temperature and viscosity at low frequency region (*ω* = 0.02 rad/s) as a function of VGCFs content for CP composites, respectively. (d) Responsivity of CP films (~50 μm) with different VGCFs contents to static saturated n-hexane vapor.

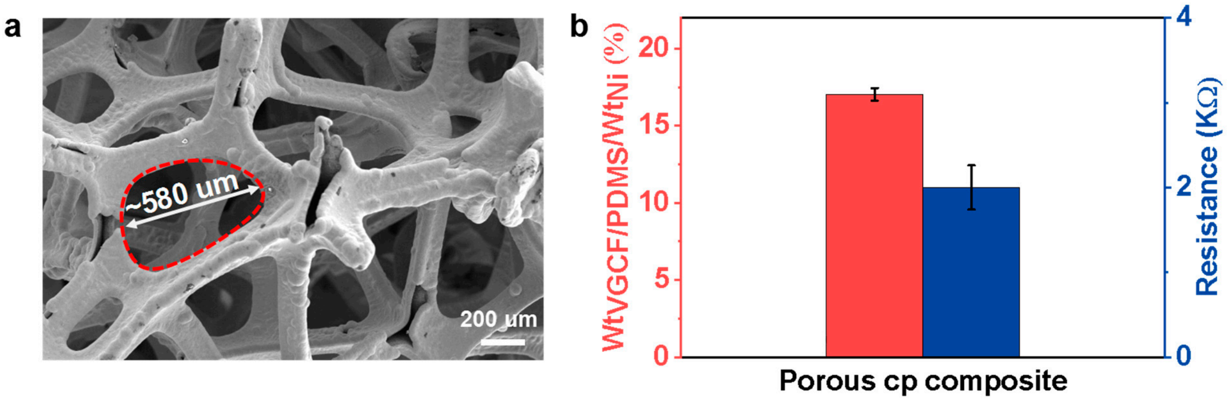

Figure S3. (a) SEM images of pristine Ni foam. (b) The weight ratio of CP coating to Ni foam and the resistance of CPS composite.

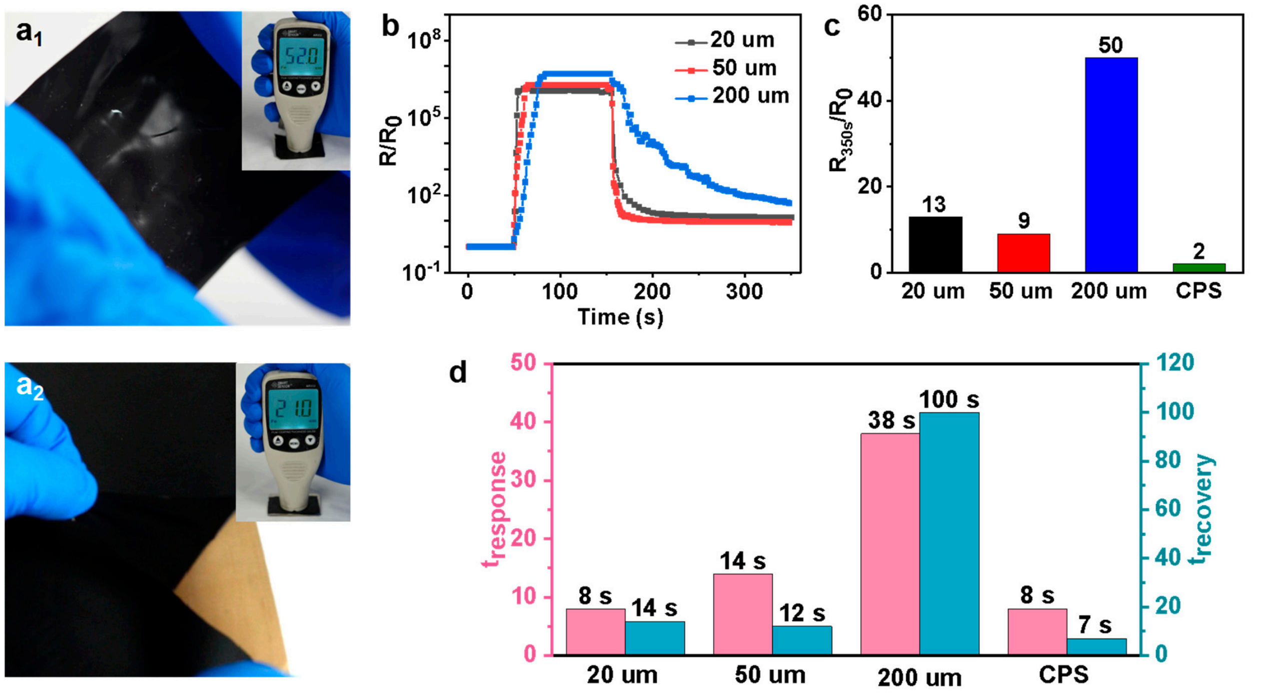

Figure S4. Photographs and corresponding thicknesses of membranes prepared by melt-moulding (a_1) and solution casting (a_2), respectively. (b) Vapor sensing behavior of VGCFs/PDMS membranes with different thicknesses to static saturated n-hexane vapor. (c) The relative resistance (R_{350}/R_0) of VGCFs/PDMS membranes with different thicknesses and CPS sample at the time of 350 s. (d) Response/recovery time of VGCFs/PDMS membranes with different thicknesses to static saturated n-hexane vapor.

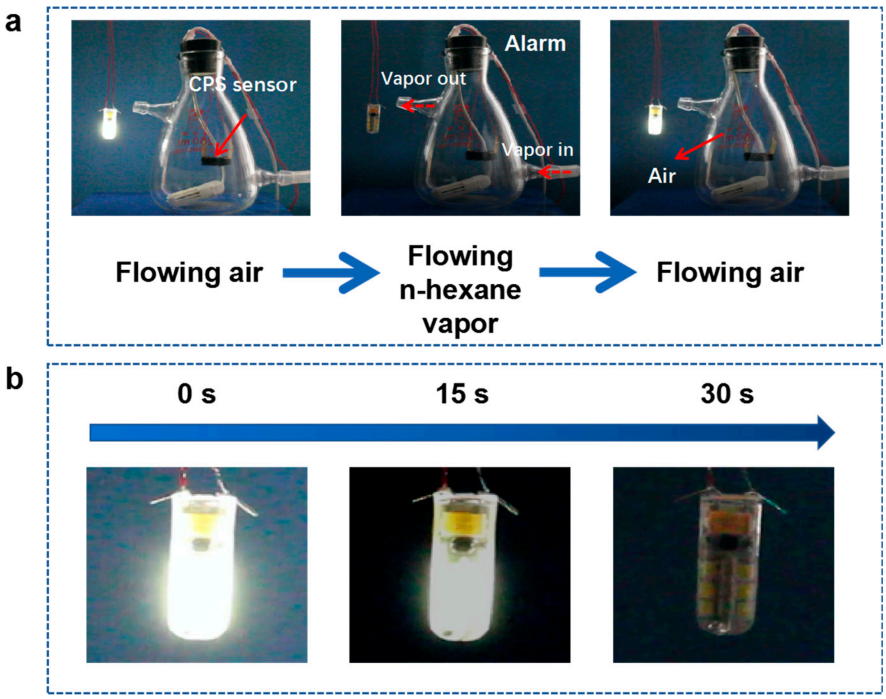

Figure S5. Photographs of simulated detection of n-hexane vapor using CPS composites and the corresponding changes in the brightness of the bulb during one cycle change of flowing vapor and flowing air (a) and in vapor flowing progress (b).

Table S1. Solubility parameters, molar volume, Flory-Huggins interaction parameters of target solvents and the corresponding response time of sensing behavior.

Target solvent	δ $(J/cm^3)^{1/2}$	Molar volume (cm^3/mol)	χ_{12}(SR-solvent)	$t_{response}$ (s)
n-Hexane	14.9	131.3	0.008	8
Carbon tetrachloride	17.6	90.6	0.203	11
Chloroform	19.0	80.7	0.440	16
Cyclohexane	16.8	106.4	0.096	18
Ethyl alcohol	26.5	57.6	2.868	/
Methanol	29.6	40.7	3.360	/

Table S2. Comparison of maximum responsivity and sensitivity among different vapor sensing materials.

Material	Maximum Responsivity	Sensitivity to saturated vapor (s^{-1})	Target organic vapors	Test condition (°C)	Reference
[a] CNTs/PEG/PU	~1.1	~ 3.6×10^{-3}	n-Hexane	Static saturated vapor	[1]
	~1.2	~ 2.2×10^{-3}	Cyclohexane		
[b] CB/RF/PLA	~3.7	~0.02	Dichloromethane	Static saturated vapor /40 °C	[2]
CNTs/RF/PLA	~2.5	~0.01	Dichloromethane		
[c] Graphene/TPU	~11.2	~0.11	Cyclohexane	Flow saturated vapor /25 °C	[3]
	~8.96	~0.09	CCl_4		
CNTs/poly(styrene-butadiene-styrene) fiber	~2×10^2	~2	Cyclohexane	Flow saturated vapor /25 °C	[4]
	~40	~0.4	Tetrahydrofuran		
CNFs/polystyrene	~6.1×10^5	~1.0×10^3	n-Hexane	Static saturated Vapor/ 35 °C	[5]
[d] VCS@PU foam	1.9×10^6	1.3×10^5	n-Hexane	Static saturated vapor/ 30 °C	[6]
[e] VS@PU foam	2.0×10^6	8.0×10^3	n-Hexane		
VGCFs/PDMS membrane (20 μm)	~1.1×10^6	~1.4×10^5	n-Hexane	Static saturated vapor 25 °C	This work
**VGCFs/PDMS sponge**	**1.2×10^6**	**1.5×10^5**	**n-Hexane**	**Static saturated vapor 25 °C**	**This work**
	**1.2×10^6**	**2.6×10^4**	**n-Hexane**	**Flow saturated vapor 25 °C**	

[a] PEG: Poly(ethylene glycol), PU: polyurethane, [b] PLA: Poly(lactic acid), RF: ramie fiber, [c] TPU: Conductive thermoplastic polyurethane, [d] VCS: vapor grown carbon nanofiber /clay/silicon rubber, [e] VS: vapor grown carbon nanofiber / silicon rubber.

Reference

1. Lee, J.; Park, E.J.; Choi, J.; Hong, J.; Metals, S.E.S.J.S. Polyurethane/PEG-modified MWCNT composite film for the chemical vapor sensor application. *Synthetic Metals* **2010**, *160*, 566–574, doi:10.1016/j.synthmet.2009.12.004.
2. Li, Y.; Liu, H.; Dai, K.; Zheng, G.; Liu, C.; Chen, J.; Shen, C. Tuning of vapor sensing behaviors of eco-friendly conductive polymer composites utilizing ramie fiber. *Sensors and Actuators B: Chemical* **2015**, *221*, 1279-1289, doi:10.1016/j.snb.2015.07.100.
3. Liu, H.; Huang, W.; Yang, X.; Dai, K.; Zheng, G.; Liu, C.; Shen, C.; Yan, X.; Guo, J.; Guo, Z. Organic vapor sensing behaviors of conductive thermoplastic polyurethane–graphene nanocomposites. *Journal of Materials Chemistry C* **2016**, *4*, 4459-4469, doi:10.1039/C6TC00987E.
4. Wang, X.; Li, Y.; Pionteck, J.; Zhou, Z.; Weng, W.; Luo, X.; Qin, Z.; Voit, B.; Zhu, M. Flexible poly(styrene-butadiene-styrene)/carbon nanotube fiber based vapor sensors with high sensitivity, wide detection range, and fast response. *Sensors and Actuators B: Chemical* **2018**, *256*, 896-904, doi:10.1016/j.snb.2017.10.028.
5. Zhang, B.; Fu, R.; Zhang, M.; Dong, X.; Wang, L.; Pittman, C.U. Gas sensitive vapor grown carbon nanofiber/polystyrene sensors. *Materials Research Bulletin* **2006**, *41*, 553-562, doi:10.1016/j.materresbull.2005.09.009.
6. Qiang, F.; Dai, S.-W.; Zhao, L.; Gong, L.-X.; Zhang, G.-D.; Jiang, J.-X.; Tang, L.-C. An insulating second filler tuning porous conductive composites for highly sensitive and fast responsive organic vapor sensor. *Sensors and Actuators B: Chemical* **2019**, *285*, 254-263, doi:10.1016/j.snb.2019.01.043.
